# Evaluation of bone-conducted sound during ear surgery: an in vivo pilot study

**DOI:** 10.1186/s12938-026-01652-0

**Published:** 2026-11-13

**Authors:** Katarina Gille, Mario Fleischer, Marcus Neudert, Thomas Zahnert, Matthias Bornitz

**Affiliations:** 1 Department of Otorhinolaryngology Head and Neck Surgery, Faculty of Medicine and University Hospital Carl Gustav Carus, TU-Dresden, Dresden, Germany; 2 Department of Audiology and Phoniatrics, Charité-Universitätsmedizin Berlin, Corporate Member of Freie Universität Berlin and Humboldt-Universität zu Berlin, Berlin, Germany

**Keywords:** Drilling noise, Ear surgery, Intraoperative monitoring, Bone conduction

## Abstract

The impact of noise induced by drilling on hearing during ear operations has been discussed several times in the past. However, direct measurements of the equivalent sound pressure level in patients undergoing ear surgery have not previously been reported. This pilot study presents an easy-to-use method based on a calibrated accelerometer to determine the equivalent sound pressure level potentially applicable in the clinical routine. During seven monitored cochlear implant surgeries, the equivalent A-weighted sound pressure level L_AF_ ranged from 111 to 126 dB(A)_eqSPL_ at its maximum, whereas C-weighted peak levels L_Cpeak_ showed peaks up to 140 dB(C)_eqSPL_. The recommended daily noise exposure limit was exceeded for three of the seven patients, suggesting the potential for drilling-induced temporal shifts in hearing threshold. Based on these findings, standardized noise monitoring is recommended as a quality assurance measure during otological surgery.

**Supplementary Information:**

The online version contains supplementary material available at https://doi.org/10.1186/s12938-026-01652-0.

## Background

Surgical drilling of the temporal bone and the skull base is an integral part of many otologic and neurotologic procedures and inevitably generates high-intensity vibratory energy that is transmitted to the cochlea via bone conduction [1]. While hearing preservation has become an increasingly important surgical goal, the order to which extent drill-induced noise contributes to cochlear injury is not completely known yet. Even limited high-frequency sensorineural hearing loss may have relevant consequences in daily life, including impaired speech perception [2]. Consequently, defining the transmission pathways, the equivalent sound pressure level (SPL), and cochlear harm caused by bone-conducted drilling noise is essential for risk assessment and the development of evidence-based strategies to minimize intraoperative auditory injury.

The potential risk of noise exposure transmitted by bone conduction during skull and ear surgery has been discussed since the 1960s. Early reports already suggest that high drilling intensities might harm cochlear structures. Schuhknecht and Tonndorf describe a postoperative threshold decline of approximately 50 dB above 2 kHz following mastoid drilling [3]. Although they conducted no measurements, they propose that drill-induced noise would generally not be harmful, except in cases of direct contact with the ossicular chain. Subsequent clinical observations support this suspicion. In a series of 1,680 operated ears, Palva et al. [4] found postoperative losses of approximately 35 dB at 4–8 kHz in 75 cases—mostly reversible—again attributing them to inadvertent ossicular contact. Helms et al. [5] later demonstrated, in temporal bone preparations, that drilling on the ossicular chain can cause stapes displacements equivalent to noise levels exceeding 130 dB SPL.

To better quantify drill-induced sound level at the cochlea, several authors performed direct physical measurements. Kylen et al. [6] measured noise levels in temporal bone and whole-skull preparations using accelerometers calibrated against a standard audiometer. They found ipsilateral cochlear exposures around 100 dB(A), suggesting potential risk particularly at high frequencies. In a follow-up study, Kylen et al. [7] varied drill size, drill type, and drilling location, concluding that none of these parameters substantially reduce bone-conducted noise; thus, limiting drilling time is the only reliable strategy to avoid noise trauma. Other studies using different measurement techniques arrived at comparable results. Kylen et al. [8] demonstrated temporary threshold shifts of 5–40 dB at 4 and 8 kHz in patients with normal hearing undergoing mastoid drilling. Palva et al. [9] similarly reported postoperative losses > 20 dB above 2 kHz, irreversible in six cases, attributed primarily to bone-conducted drill noise. Holmquist et al. [10], using subjective loudness matching during local anaesthesia, found peak levels up to 130 dB(A) SPL depending on drill type, further supporting a possible risk to the cochlea. Parkin et al. [11] measured airborne sound in the middle and external ear during mastoid drilling and reported average levels around 107 dB(A) SPL, complementing earlier bone-conduction measurements. In a study involving 63 patients who underwent mastoidectomy, Neeraj et al. [12] concluded that cutting drills and large-diameter drills generate more noise during drilling. Gille et al. [13] also concluded that about one-third of the variability in drilling depends on the parameters cutting speed, diameter, and applied force. Each of these parameters contributes to almost the same extent. Similarly, Kumar et al. [14] summarize that, in addition to being a source of noise, the drill is also a significant source of vibrations transmitted to the cochlea. Therefore, they conclude that surgeons should select appropriate drills to minimize vibrations in the temporal bone.

The effect on the contralateral ear has also been investigated. In a study with 62 patients, Man and Winerman [15] observed only mild ipsilateral sensorineural losses in 14 patients with no measurable effect on the contralateral ear. Spencer and Reid [16] confirmed these findings, showing that air- and bone-conducted components during middle ear surgery reach similar magnitudes (up to 100 dB(A) SPL for short periods). Using high-frequency bone-conduction audiometry, Doménech et al. [17] found postoperative losses in 9 of 24 patients, suggesting that high-frequency analysis may reveal effects not detected in standard audiometry. Tos et al. [18], however, reported no average contralateral impairment in 50 patients after translabyrinthine surgery, though individual cases show hearing threshold shift up to 20 dB. In contrast, the study by Perez et al. [19] shows that 40.8% of patients experienced a loss of 10 dB or greater and a deterioration in their hearing at least one frequency post-operatively in the contralateral, unoperated ear. Amiri et al. [20] also assume that contralateral hearing loss following skull base and middle ear surgeries is possible.

Using electrocochleography during mastoidectomy, Hickey et al. [21] recorded drill-induced cochlear levels at around 90 dB (HL) and concluded that significant postoperative impairment is unlikely. Similarly, Urquhart et al. [22] found no significant hearing threshold changes in 40 patients.

Animal studies, however, show variable outcomes. Suits et al. [23] observed fully reversible threshold shifts from 2 to 32 kHz in guinea pigs, suggesting temporary effects. In contrast, Gjuric et al. [24] found irreversible losses of 20–70 dB when drilling directly at the ossicular chain. This is consistent with earlier mechanical findings by Helms et al. [5] on human specimens.

Comparative analyses of different surgical instruments have also been performed. Michaelides et al. [25] measured airborne noise during temporal bone drilling and reported peak levels up to 132 dB(A) SPL for pneumatic drills and erbium lasers, indicating a substantial risk for postoperative cochlear damage. In contrast to most prior work focusing on air conduction thresholds, Hallmo et al. [26] examined high-frequency bone-conduction thresholds (up to 16 kHz) pre- and post-operatively in 45 patients and found no significant changes.

Further insight comes from studies quantifying excitation acceleration and exposure time. Sutinen et al. [27], building on the approach by Zou et al. [28], measured acceleration levels (4.2–18.8 m/s^2^) and exposure durations (15 min) that produced temporary threshold shift up to 10 dB between 500–1000 Hz in guinea pigs, which recovered within 7 days. In clinical follow-ups using audiometry and otoacoustic emissions, Karatas et al. [29] show that all postoperative changes disappear within 72–96 h. However, Migirov et al. [30] reported persistent distortion product otoacoustic emissions amplitude reductions at 2–4 kHz in some patients one month after surgery, and Farzanegan et al. [31] likewise found permanent sensorineural losses up to 10 dB at 4–6 kHz.

While most studies report no significant long-term impairment on average, multiple authors document individual cases with irreversible threshold shifts—especially at high frequencies or when ossicular contact occurs. This variability was already highlighted by Kylen et al. [6], who emphasized that reliable data must come from experiments on intact skulls, as vibratory patterns differ substantially between isolated temporal bones and the complete skull.

To quantify bone-conducted vibrations generated during in vivo surgical drilling under conditions representative of the intact human skull, a dedicated measurement system suitable for measurements in the operating room was developed and implemented. The focus of this investigation was on the feasibility of the measurement system and raised the question of whether the critical noise dose is exceeded during surgery and whether differences in the noise generated by the drill can be detected during standardized ear surgeries.

## Material and method

### Patients and surgical system

The study followed the ethical principles established by the WMA Declaration of Helsinki and was approved by the ethics committee of Universitätsklinikum Carl Gustav Carus Dresden (EK150042011). All patients provided informed consent. The number of participants was deliberately limited to the necessary minimum. As our study was conducted in the context of clinical surgical procedures, it was essential to avoid any disruption to routine surgical procedures to minimize risks and stress and not to interfere with them. For this study, seven patients (3 male/4 female, median age 69 years, interquartile range 23 years), undergoing cochlear implant surgery, were monitored. These surgeries are highly standardized, with a low complication rate, making them a reliable and safe procedure [32]. The uniformity of the surgical technique ensures comparable measurement results across surgeries performed on different patients.

The seven operations were performed by three different surgeons (one surgeon per surgery) with a comparable level of experience, using five different types of surgical drills (see Fig. 3) for the individual steps during the surgery. Notably, one out of three surgeons underwent five surgeries, whereas the other surgeons underwent only one surgery. Furthermore, not all surgeons used the same number of surgical drills. Unfortunately, it was not possible to monitor the rotational speed of the surgical drills during the surgeries. Moreover, the standard surgical procedure was maintained during the measurements.

### Measurement system and data collection

**Fig. 1:**
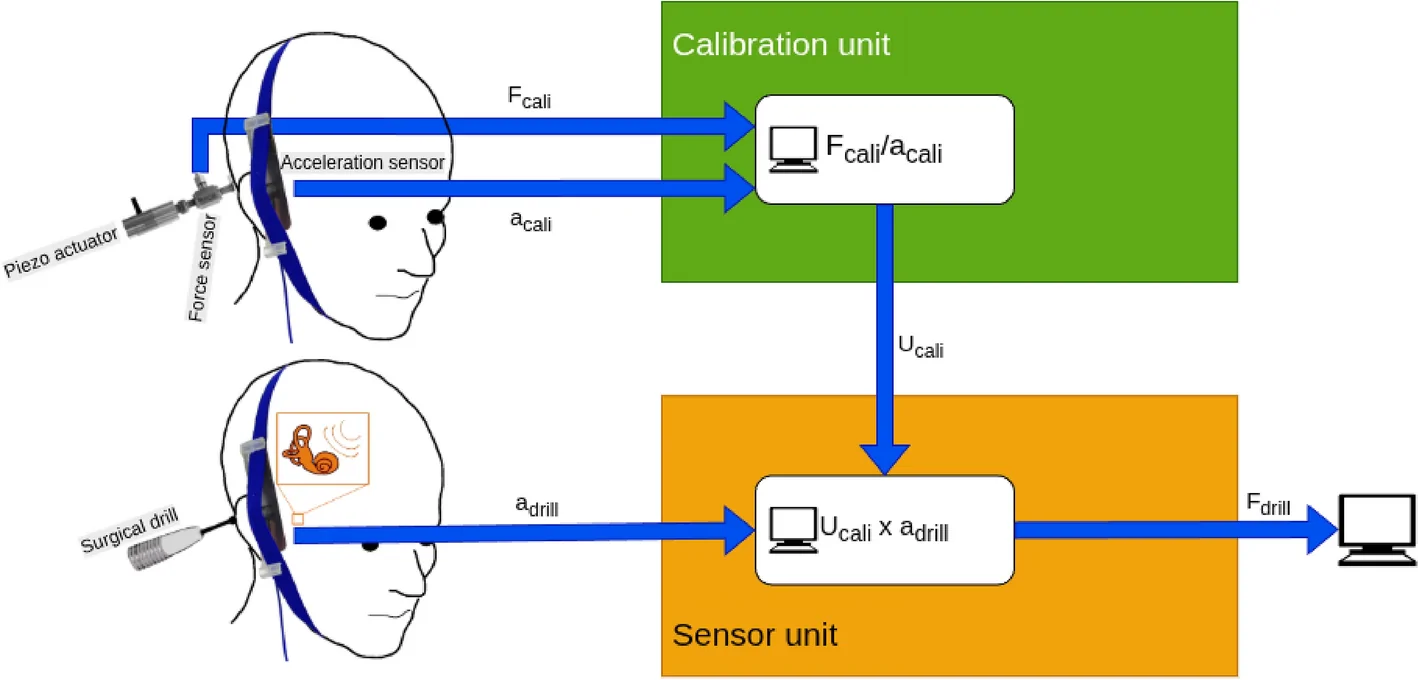
Scheme of the calibration (green area) and sensor unit (orange area). The data pipeline consists of a patient-specific preoperative calibration (U_cali_ = F_cali_/a_cali_) and data acquisition during surgery (F_drill_ = U_cali_ x a_drill_)

The measurement system consisted of the following components: calibration unit (consisting of a piezo actuator (P-840.1, PI Physik Instrumente, Karlsruhe, Germany), a force sensor (209C11, PCB Piezotronics, Depew, NY, USA), and a handle (self-constructed)), a sensor unit (consisting of an acceleration sensor (vibration sensor in TO5 housing, PCB Piezotronics, Depew, NY, USA) and an elastic band), the measurement device and a processing unit (NI USB-4431, National Instruments Austin, TX, USA, LabView-Program). Parts of the system are shown schematically in Fig. 1.

Before surgery, but before the patient was covered and before the surgical area was sterilized, the sensor unit was calibrated by applying a defined force *F*_*cali*_ to the mastoid of the anaesthetized patient via the calibrator unit. Therefore, the calibrator unit, similar to a bone conduction receiver (DIN EN ISO 389-3 [33]), was pressed on the skin, covering the mastoid, by hand with a static contact force of about 5 N. Here, to comply with the coupling area specified in DIN En 60318-6 [34] (175 mm^2^ ± 25 mm^2^), the use of a handle was necessary. A multi-sinus with a bandwidth of 6 kHz, frequency increment of 5.86 Hz and quadratic phase (second-degree polynomial) was applied via the piezo actuator (see Fig. 1). With the force sensor (see Fig. 1), connected in series, the calibration force (*F*_*cali*_) was measured directly. Simultaneously, the sensor unit records the acceleration *a*_*cali*_. Finally, the frequency response *U*_*cali*_ = *F*_*cali*_*/a*_*cali*_ was stored individually for each patient. The calibration measurement (always the complex valued mean of ten repetitions per trial was considered) was repeated, if necessary, until most of the frequency response (consisting of real and imaginary parts, i.e. phase references were retained) had a spectral coherence (C, see [35], Eq. 5.90) greater than 0.9, resulting in a normalized standard error of *U*_*cali*_ lower than 0.075 (see [35], Eq. 9.90). Frequency ranges where the spectral coherence was less than 0.9 were excluded from analysis. During the whole surgery, the sensor unit remained in place. Therefore, an elastic band was used to fix the acceleration sensor at the patient's skull in such a way that medical care (e.g., ventilation) is not disrupted and constant contact pressure was maintained. During surgery, the drilling force *F*_*drill*_, exerted by the surgical drill at and close to the calibration point, was determined by blockwise (blocksize 2048 samples) multiplying the acceleration *a*_*drill*_ by *U*_*cali*_.

### Data post-processing

After each surgery, the final evaluation, including the calculation of cumulative noise dose due to the noise exposure, was performed semi-automatically on the basis of the measured data. In standards and other studies noise exposure is usually related to air conduction (AC) with equivalent continuous or peak sound pressure levels as characteristic exposure values. The drilling force *F*_*drill*_ was therefore converted to an equivalent SPL. According to DIN EN ISO 389-3 (for BC [33]) and DIN ISO 226 (for AC and free-field condition [36]), the reference equivalent force and pressure levels, as well as their differences, depend on the frequency. Therefore, *F*_*drill*_ (in N) was filtered by a third-octave-band filter at centre frequencies *f*_*c*_ ranging from 250 to 5000 Hz to cover the frequency range up to 6 kHz (without overlapping). Further, for each frequency band, the equivalent acoustic pressure *p*_*drill*_ was calculated as *p*_*drill*_*(f*_*c*_*)*=*F*_*drill*_*(f*_*c*_*)⋅10*^*((RETSPL−RETFL)/20*^*⋅20*, where *RETSPL* and *RETFL* are the reference equivalent threshold sound pressure level (DIN ISO 226, [36]) and force level (DIN EN ISO 389-3, [33]) at *f*_*c*_. For each frequency band, *p*_*drill*_*(f*_*c*_*)* was either A-filtered and fast-time weighted (to get *L*_*AF*_(*f*_*c*_) in dB(A)_eqSPL_) or C-filtered only to get the peak value *L*_*Cpeak*_(*f*_*c*_) in dB(C)_eqSPL_, respectively. Finally, to get overall levels *L*_*AF*_ and *L*_*Cpeak*_, these quantities were computed to ${L}_{AF}=10\times\mathit{log}\left(\sum_{i}{10}^{\frac{{L}_{AF}\left({f}_{c,i}\right)}{10}}\right)$(equivalent for *L*_*Cpeak*_(*f*_*c*_)).

Furthermore, in accordance with [37], the maximum recommended noise exposure levels were calculated based on the time-integrated effective sound pressure levels. To consider potentially harmful doses of equivalent sound pressure levels, only values greater than 85 dB(A) SPL were taken into account [37]. These values can be clearly attributed to the surgical drilling. Various magnetic fields in the operating room and bipolar coagulation generated interference signals that were scattered into the measurement equipment. These signals constituted measurement artefacts rather than actual acoustic emissions and remained below 85 dB(A) SPL.

For statistical analysis, linear models were built within the freely available software R (v.4.6.1, https://www.r-project.org/). Here, the grouped medians (grouped for surgical drill, the surgeons’ ID (SID), the patients’ ID (PID), and sex) were used, whereas, because of highly unbalanced data, only the surgical drill and sex were used as the independent variables (all three variables were considered as categorical variables), and *L*_*AF*_ (numerical value) were used as the dependent variable. To evaluate whether each of the independent variables had an impact on *L*_*AF*_, a one-way ANOVA (type III, because of unbalanced data) of the models was used. For all models, residual plots and Q–Q plots were carefully visually inspected to preclude strong deviations from homoscedasticity and normality (see Figure S2). Because of the low sample size, considering SID as a random effect, within a linear mixed effects model, was not considered.

## Results

### Calibration of the measurement setup

**Fig. 2:**
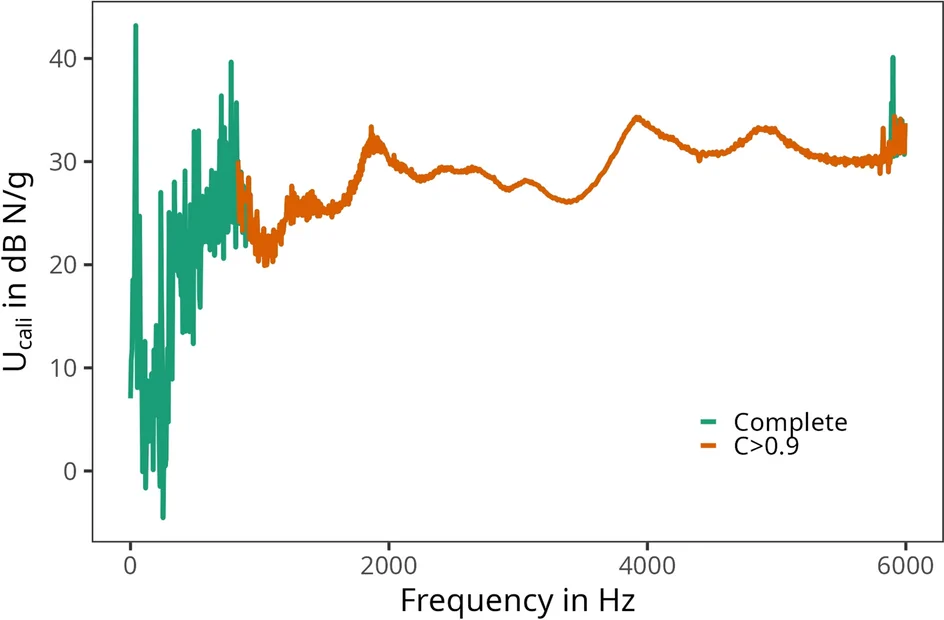
Magnitude of *U*_*cali*_, exemplified by patient PID7. Green colour denotes the complete curve, and orange colour denotes the region where the spectral coherence *C* was greater than 0.9

As it was not possible to measure the equivalent sound pressure level perceived by the anaesthetized patients directly, using sensor calibration of an accelerometer performed beforehand was assessed. To achieve adequate quality, only frequency components with spectral coherence *C* > 0.9 were considered from the frequency responses *U*_*cali*_ of the calibration. An example of *U*_*cali*_, including the frequency range considered, is shown in Fig. 2 (see Figure S1 for all patients). The quality of *U*_*cali*_ depends on the specific conditions. That demonstrates that frequencies below 1 kHz typically exhibit low spectral coherence and consequently, a low signal-to-noise ratio. For frequencies higher than 1 kHz, the spectral coherence indicated a good quality of *U*_*cali*_. Notably, the calibration was only done before surgery.

### Effect of surgical drill, sex, and surgeon on equivalent sound pressure level

**Fig. 3:**
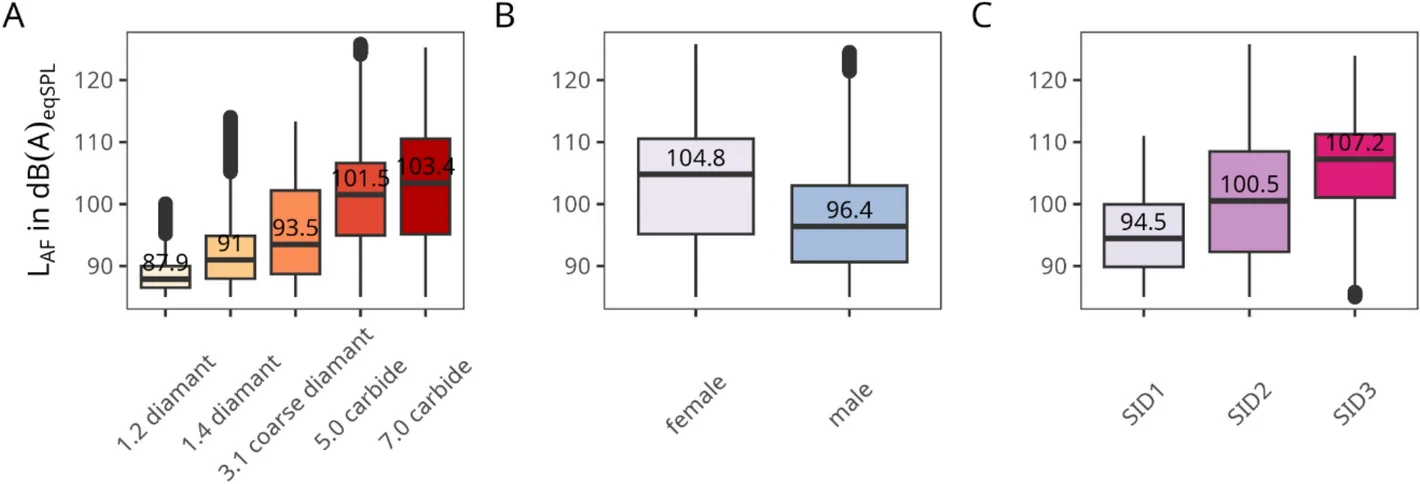
**A** Equivalent sound pressure levels *L*_*AF*_ determined depending on the surgical drill, **B** the patients’ sex, and **C** the surgeon. Numbers denote the median values of *L*_*AF*_ in dB(A)_eqSPL_

The *L*_*AF*_ values varied depending on the surgical drill used (Fig. 3A). The larger the diameter of the surgical drill, the greater the *L*_*AF*_. Whereas the smallest diamond drill (diameter 1.2 mm), mostly used for the cochleostomy, led to values around 87.9 dB(A)_eqSPL_, the large carbide drill (7 mm, used for the preparation of the implant bed) led to values around 103.4 dB(A)_eqSPL_ with peaks in the order of 125 dB(A)_eqSPL_. Additionally, a dependency on the diameter of the surgical drill and L_AF_ was confirmed by linear modelling. The surgical drill significantly influenced *L*_*AF*_ (*p* < 0.0001, see Table S6). Relative to the 1.2 mm diamond drill, the 1.4 mm diamond drill produced 2.3 ± 2.7 dB (standard errors, *p* = 0.40, not significant) higher *L*_*AF*_-values, the 3.1 coarse diamond drill produced 7.2 ± 2.7 dB (*p* = 0.015, significant) higher L_AF_-values, the 5.0 carbide drill produced 16.8 ± 2.6 dB (*p* < 0.0001, significant) higher *L*_*AF*_-values, and the 7.0 carbide drill produced 16.1 ± 2.5 dB (*p* < 0.0001, significant) higher *L*_*AF*_-values, respectively (see Table S7).

*L*_*AF*_ was influenced by sex (Fig. 3B). Descriptively, the median *L*_*AF*_ for females was 104.8 dB(A)_eqSPL_, whereas for males it was 96.4 dB(A)_eqSPL_. Linear modelling confirms that sex significantly influenced *L*_*AF*_ (*p* = 0.001, see Table S6). Based on the statistical model, in females, *L*_*AF*_-values were 6.1 ± 1.6 dB (*p* = 0.001, significant) higher than in males (see Table S7). However, because of the low sample size, sex differences should be interpreted cautiously.

Although all of the surgeries examined were performed by experienced surgeons, there were also differences between them (Fig. 3C). The median *L*_*AF*_ ranged from about 94.5 dB(A)_eqSPL_ (SID1) up to 107.2 dB(A)_eqSPL_ (SID3), which could, because of highly unbalanced data, not be proven via linear modelling.

**Fig. 4:**
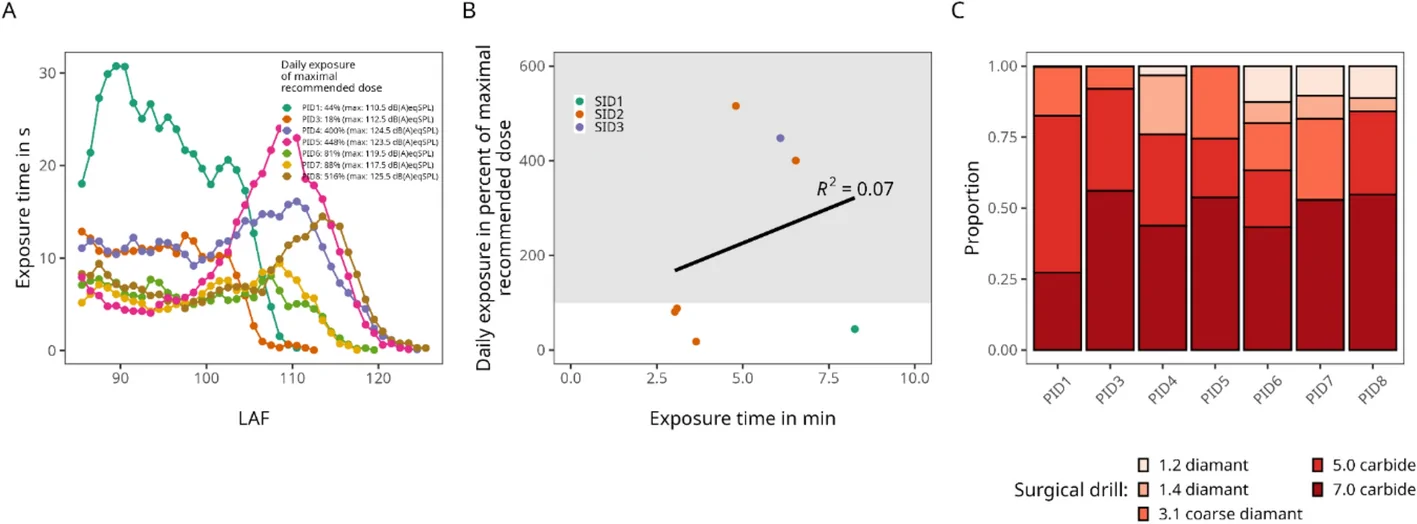
**A** Exposure times as a function of the equivalent sound pressure level *L*_*AF*_ for all patients (coloured by the ID of the patient (PID)), including cumulative daily dose of noise exposures and maximum of *L*_*AF*_ as shown in the legend. **B** Cumulative daily dose as a function of exposure time for each patient (denoted by points) and coloured by the ID of the surgeon (SID) as denoted in the legend. **C** Proportions of specific surgical drill (as denoted in the legend) for each patient on drilling time with equivalent sound pressure levels greater than 85 dB(A) SPL

Further analysis showed that the permissible daily dose, according to Chan et al. [37], was exceeded in three of the seven operations (Fig. 4A). Here, up to 516% (PID8) of the maximal daily dose was observed, whereas the maximal value of *L*_*AF*_ was in the range of 110.5 dB(A)_eqSPL_ (PID1) and 125.5 dB(A)_eqSPL_ (PID8). In addition to *L*_*AF*_, *L*_*Cpeak*_ was also calculated to determine short-term peak levels. Depending on the patient, *L*_*Cpeak*_ was in the range of 124 dB(C)_eqSPL_ (PID3) to 140 dB(C)_eqSPL_ (PID4) (see Table S3).

There was only a weak correlation (R2 = 0.07, linear regression, see Fig. 4B) between the exposure time (drilling time) and the noise dose administered (Fig. 4B). Although there was a trend showing that the longer the drilling time, the higher the daily dose, the linear relationship was not statistically significant. In Fig. 4C, the relative proportion of time spent by each surgical drill during the respective operation is demonstrated. The amount of time of the equivalent sound pressure levels greater than 85 dB(A) SPL of surgical drills with a large diameter was notably higher than for those with a small diameter. Notably, only sound levels above 85 dB(A)_eqSPL_ were taken into account in this study. This threshold corresponds to the 8-h time-weighted average exposure level defined by established hearing-damage standards (NIOSH [37], Iso 1999 [38]), below which noise exposure is generally not considered to pose a risk of noise-induced hearing loss at the population level. Nevertheless, the 85 dB(A) criterion was adopted here to ensure comparability with occupational damage-risk frameworks, which are likewise used as the basis for noise-limiting regulations at music events (e.g., DIN 15905-5 [39]). Furthermore, it should be noted that the underlying damage-risk models are based on a typical weekly working pattern of five eight-hour exposure days, whereas the present measurements represent a single exposure event.

## Discussion

This article presents an easy-to-use and robust method for determining the equivalent sound pressure level and noise exposure dose from drilling during ear surgeries. The study demonstrates the applicability of the method in a clinical setting and provides initial findings on the impact of various parameters (drill type, patient sex, and surgeon) on noise exposure. The presented method provides a good approximation of the hearing impression, expressed as equivalent sound pressure level, through bone conduction compared to the hearing impression through air conduction as proposed by [11, 40], without relying on a measurement in the inner ear [1]. That was achieved by shifting the loudness curves for bone conduction to the loudness curves for air conduction. However, loudness compression was not taken into account.

### Calibration of the measurement setup

The basis of the presented method was the calibration achieved by a single-axis accelerometer applied on the skin outside the surgical field, measuring the force signal of a piezo-actuator at the mastoid. A single-axis accelerometer was used to measure the vibration in the predominant direction, normal to the skull bone surface [41] and on the mastoid close to the cochlea [42], corresponding to bone-conducted stimulation. In general, 3D accelerations occur in the skull bone during bone conduction stimulation. However, experimental studies have shown that, for ipsilateral measurements, uniaxial and triaxial recordings yield comparable results [41, 43].

Initial drilling during cochlear implant (CI) surgeries is performed at the mastoid (mastoidectomy, implant bed), which is also the location closest to the inner ear. Considering a normalized standard error of *U*_*cali*_ of 0.075, the 95% confidence interval was between (|*U*_*cali*_|−1.2) dB N/g and (|*U*_*cali*_| + 1.4) dB N/g (see [35], Eq. 8.13), indicating that the determination of *U*_*cali*_, and therefore the calibration, is proved to be highly accurate and reliable, but because of noise in the estimation, limited to a frequency of around 1 kHz up to 6 kHz. This range corresponds to the most critical frequency range of human hearing [44], whereas at frequencies below 1 kHz, the hearing threshold is significantly higher. The insufficient calibration quality below 1 kHz is primarily due to the design of the calibration. Due to the free-oscillating construction of the piezo actuator, marginal inertial mass can be applied at low frequencies, resulting in a low signal-to-noise ratio. The extent to which a tree-axial accelerometer can overcome these limitations was beyond the scope of this study due to the lack of commercially available supplies with adequate sensitivity.

### Effect of surgical drill, sex, and surgeon on equivalent sound pressure level

It could be proven that larger drills resulted in higher noise levels in accordance with Neeraj et al. and Kumar et al. [12, 14]. That seems plausible, because drills with larger diameters are specifically used for the hard cells of the skull, and noise exposure caused by bone conduction is therefore likely to be particularly strong. That finding is consistent with that of Prodanovic et al. [45]. However, it must be taken into account that the cutting speed is not constant at a given rotational speed across different surgical drills. Further, the rotational speed is typically higher with large-diameter surgical drills than with small-diameter ones, which are typically used near the cochlea. Since all measured sound levels refer to the calibration performed before the skin incision, deviations from the calibration point are expected to increase over the course of the operation. Consequently, the drilling noise generated when using small-diameter drills near the cochlea was (likely) underestimated due to the increasing deviation between the drilling location and the calibration position, as well as changes in bone structure following mastoidectomy. Regarding changes in bone conduction depending on the stimulation position, the results align with [46], which suggests that a stimulation position close to the cochlea for BC hearing aids is beneficial. In sum, the combined effect of calibration, drilling location, and rotational speed leads to an accumulation of errors, resulting in an underrating of the levels especially when using small drills.

Higher noise exposure was observed in females than in males. This result seems surprising, as females are expected to have lower bone density due to a higher likelihood of developing osteoporosis [47]. It should be noted, however, that the four females studied may not have been representative. Furthermore, it is conceivable that the calibration of the measurement system should be sex-specific. However, those issues could not be fully addressed in this study and could be the subject of further research.

Differences of more than 12 dB were observed between the results for supervised surgeons. Unfortunately, due to the unbalanced dataset (two of the three surgeons were supervised once), no clear statistical trend between the surgeons could be derived. Although all procedures were performed by experienced surgeons, the surgeon’s own drilling technique influenced the noise exposure during drilling. Additionally, patients’ own individual factors, i.e. their anatomy, bone density, and dampening by surrounding tissue, could not be addressed because of the unique surgeon–patient combination.

### Noise exposure during surgery

While the median *L*_*AF*_ values, depending on drill type, sex, and surgeon, ranged from 87.9 to 107.2 dB(A)_eqSPL_ (see Fig. 3), the individual maximum *L*_*AF*_ values ranged from 110.5 to approximately 125.5 dB(A)_eqSPL_ (see Fig. 4A), and the *L*_*Cpeak*_ reached up to 140 dB(C)_eqSPL_. These values thus confirm the values determined in previous studies [6, 10, 11]. For comparison: Kujawa et al. [48] selected in their study of ‘Cochlear Nerve Degeneration after “Temporary” Noise-Induced Hearing Loss’ with mice, a deliberate acoustic overexposure stimulus of 100 dB SPL for 2 h. They concluded that even if hearing thresholds appear to recover following exposure to noise, irreversible damage occurs to the nerve fibres of the cochlea.

Furthermore, it was found that the distribution of exposure time as a function of *L*_*AF*_ varied greatly among individual patients. So, the maximum recommended noise dose was not reached or exceeded in all patients. In this study, the noise dose ranged from 18% (PID3) to 516% (PID8) of the maximum recommended noise dose, being exceeded in three out of seven surgeries (see Fig. 4A). Therefore, following ear surgery, a (temporary) shift in the hearing threshold could occur in individual cases [4, 8], which, due to mechanical vibrations of the entire skull, need not be limited to the ipsilateral side [15, 16, 19, 20].

However, the relationship between exposure time and noise dose was not significant. That could be related to the choice of surgical drill, and thus to individual physiological factors such as bone density, cell structure, and specific anatomy (see Fig. 4B and C). Therefore, selecting surgical drills with smaller diameters alone does not necessarily result in a lower noise dose [10, 12, 14].

However, it seems that a longer drilling duration is more likely to result in the permissible daily noise exposure limit being exceeded than short-term peak levels. Contrary to expectations, this would suggest that short-term, high-intensity drilling is preferable to extending the drilling duration. Due to the differing approaches and methodologies used in studies on noise exposure during drilling (see Background), a direct comparison of the measured noise levels between the results of other studies and those of the present study is not possible.

### Limitations and future work

The number of participants was deliberately limited to the necessary minimum, as the measurements were taken as part of routine clinical practice. Therefore, the resulting small sample size and the unbalanced data should be considered as proof of concept.

A reduction in measurement accuracy is expected when drilling on delicate structures such as the cochlea (e.g., during cochleostomy), as the applied calibration does not account for changes in local mechanical coupling. However, this effect cannot be determined conclusively based on the limited data presented. Alternative measurement approaches, such as electrophysiological methods or intracochlear pressure measurements [1], could potentially quantify these effects more precisely. However, their intraoperative application and/or technical implementation are not yet well established [49].

The calibration accuracy decreases at frequencies below 1 kHz due to a poor signal-to-noise ratio. These frequency regions were therefore removed to ensure data quality and reliability, resulting in systematic underestimation of the drilling-induced noise levels. To overcome this limitation, it is necessary to improve the calibration method below 1 kHz to extend the usable frequency range to the fundamental frequency of the surgical drill (e.g., 333 Hz @ 20,000 rpm).

The frequency spectrum of the drill encompasses at least the investigated frequency range due to the presence of higher harmonics [50], indicating that the applied calibration captures the majority of the mechanical energy introduced by the surgical drill. Consequently, the measured values represent a lower bound of the equivalent sound pressure level. Further, due to the small sample size, this study was unable to fully assess the interaction between the surgical drill, sex, and surgical handling.

In sum, it can be concluded that during ear surgeries, cumulative noise exposure and peak noise levels may be harmful even with short exposure (see [37]). However, intraoperative drilling noise occurs in a broader context. It is also important to consider the noise generated by mechanical work on the skull in oral and maxillofacial surgery, neurosurgery, and plastic surgery not only for the patient, but also for the surgeon [51].

Future studies should therefore assess individual consequences in larger cohorts, determining pre- and postoperative audiometry and follow-up while taking into account drill type, sex, patients’ age, morphological differences and factors, and the surgeon's experience and skill. The potential for hearing damage should be monitored simultaneously during ear surgeries. Then, immediate action could be taken to avoid exceeding limits that could harm patients' hearing ability.

## Conclusion

In summary, the presented measurement procedure is easily integrable into routine surgical practice without a large extension of the surgery. Furthermore, our results indicate that the permissible daily noise dose is demonstrably exceeded during the surgical procedure. Based on these findings, standardized noise monitoring during otologic surgery is recommended and could be a measure for quality assurance.

## Supplementary Information

Additional file 1.

## Data Availability

All measured data and R-scripts used for analysis, computation and visualization are available in the Supplementary information.

## Abbreviations

AC: Air conduction
BC: Bone conduction
C: Coherence
CI: Cochlear implant
TTS: Temporary Threshold Shift
RETFL: Reference Equivalent Threshold Force Level
